# Implementing a new physiotherapist-led primary care model for low back pain: a qualitative study of patient and primary care team perspectives

**DOI:** 10.1186/s12875-022-01817-5

**Published:** 2022-08-11

**Authors:** Kyle Vader, Catherine Donnelly, Simon D. French, Colleen Grady, Jonathan C. Hill, Dean A. Tripp, Ashley Williams, Jordan Miller

**Affiliations:** 1 School of Rehabilitation Therapy, Queen’s University, Louise D Acton Building, 31 George St, Kingston, ON K7L 3N6 Canada; 2 Department of Chiropractic, Macquarie University, Sydney, Australia; 3 Centre for Studies in Primary Care, Queen’s University, Kingston, Canada; 4 School of Medicine, Keele University, Staffordshire, UK; 5 Departments of Psychology, Anesthesiology, & Urology, Queen’s University, Kingston, Canada

**Keywords:** Low back pain, Primary care, Physiotherapy, Interviews, Focus groups, Qualitative research

## Abstract

**Background:**

Low back pain (LBP) is one of the most common reasons for primary care visits and is the leading contributor to years lived with disability worldwide. The purpose of this study was to understand the perspectives of patients and primary care team members related to their experiences with a new physiotherapist-led primary care model for LBP.

**Methods:**

We conducted an interpretive description qualitative study. Data were collected using a combination of semi-structured interviews and focus group discussions and analyzed using thematic analysis. Participants included adults (> 18 years of age) with LBP and primary care team members who participated in a physiotherapist-led primary care model for LBP in Kingston, Ontario, Canada.

**Results:**

We conducted 18 semi-structured interviews with patients with LBP (10 women; median age of 52) as well as three focus group discussions with a total of 20 primary care team members representing three teams. Four themes (each with sub-themes) were constructed: 1) enhanced primary care delivery for LBP (improved access and engagement in physiotherapy care, improved communication and care integration between the physiotherapist and primary care team, less inappropriate use of healthcare resources); 2) positive patient experiences and perceived outcomes with the new model of care (physiotherapist built therapeutic alliance, physiotherapist provided comprehensive care, improved confidence in managing LBP, decreased impact of pain on daily life); 3) positive primary care team experiences with the new model of care (physiotherapist fit well within the primary care team, physiotherapist provided expertise on LBP for the primary care team, satisfaction in being able to offer a needed service for patients); and 4) challenges implementing the new model of care (challenges with prompt access to physiotherapy care, challenges making the physiotherapist the first contact for LBP, and opportunities to optimize communication between the physiotherapist and primary care team).

**Conclusions:**

A new physiotherapist-led primary care model for LBP was described by patients and primary care team members as contributing to positive experiences and perceived outcomes for patients, primary care team members, and potentially the health system more broadly. Results suggest that this model of care may be a viable approach to support integrated and guideline adherent management of LBP in primary care settings.

**Supplementary Information:**

The online version contains supplementary material available at 10.1186/s12875-022-01817-5.

## Background

Low back pain (LBP) is the leading contributor to years lived with disability [1] and lost work productivity [2]. LBP is among the most common reasons for family physician visits [3–5]. LBP is also costly to healthcare systems due to a large number of unnecessary specialist consultations and advanced diagnostic procedures [6, 7]. People with LBP incur approximately 60% higher healthcare costs than those without LBP [8]. Recent literature has called for action to move away from biomedical and fragmented models of care for LBP [9]. Given the growing burden of LBP, innovative and evidence-based models of care are needed to improve patient outcomes and experiences, improve health system efficiency, and reduce the unnecessary use of potentially harmful and ineffective diagnostics and interventions [9–11].

Primary care has been identified as a core element of health system sustainability [12]. Primary care services focus on the treatment of a broad range of health conditions, with a focus on health promotion and disease prevention [13]. The concept of a Patient’s Medical Home, which describes new ways of organizing primary care services, has received growing attention across multiple health systems and is the vision for the future of primary care in Canada [14–16]. Unlike traditional primary care models which are focused on care provided solely by family physicians, a Patient’s Medical Home includes a diverse interprofessional healthcare team who may not all be co-located but provide connected care [17]. One of the foundations of the Patient’s Medical Home is team-based care [17]. Team-based primary care includes a broad range of services delivered by an interprofessional team where a patient does not always see their family physician, but interacts with the most appropriate member of the primary care team [18].

Physiotherapists are increasingly being integrated into primary care teams and bring particular expertise in the management of chronic diseases and musculoskeletal conditions [19–21]. Although previous research has evaluated models of care that include a primary care visit followed by early referral to a physiotherapist [22, 23], there is a dearth of research on models of care where a physiotherapist is integrated directly within the primary care team for patients with an unmet need for treatment, and available as a first point of contact for LBP. Emerging evidence has explored perspectives toward first-contact primary care physiotherapy for patients with musculoskeletal disorders and LBP in some contexts, such as the United Kingdom [24–26]. However, similar models have not yet been explored in the Canadian context.

We implemented a new physiotherapist-led primary care model for LBP as part of a pilot cluster randomized controlled trial [27]. In this model of care, a physiotherapist was integrated directly within a primary care team and available as the first point of contact who could provide additional care for patients with LBP and an unmet need for treatment. Before implementing a fully powered trial, a thorough understanding of patient and primary care team perspectives was needed to understand acceptability and inform future iterations to the model of care. The purpose of this study was to understand the perspectives of patients and primary care team members related to their experiences with a new physiotherapist-led primary care model for LBP.

## Methods

### Design

We conducted an interpretive description qualitative study [28]. Interpretive description aims to “provide a thematic or integrative description of a phenomenon of applied practical interest” [28]. We chose an interpretive description approach as it is well suited for qualitative research within the context of healthcare that has practical application [28]. This study was nested within a pilot cluster randomized controlled trial that set out to determine the feasibility of implementing the new physiotherapist-led primary care model for LBP [27].

### Theory and framework

This research was situated within a constructivist worldview, where we approached the data inductively and acknowledged that multiple realities may simultaneously exist [29]. We broadly conceptualized primary care using concepts presented within the Patient’s Medical Home [17].

### Research team

Our research team included a physiotherapist and PhD candidate (KV), occupational therapist and researcher (CD), researcher with a background in chiropractic (SF), research manager and researcher (CG), physiotherapist and researcher (JH), health psychology professor and researcher (DT), occupational therapist and PhD candidate (AW), and physiotherapist and researcher (JM).

### Setting

Participants were recruited from interprofessional primary care teams (i.e., Family Health Teams [30] and Community Health Centres [31]) in Kingston, Ontario, Canada that participated in the new physiotherapist-led primary care model for LBP as part of a pilot cluster randomized trial [27].

### Model of care

In the new model of care, physiotherapists were integrated within primary care teams and able to deliver first contact care with no out-of-pocket costs to patients with LBP [27]. In the pilot study, patients who called to book an appointment with their family physician at their primary care site were invited to see a physiotherapist as their first point of contact if their primary reason for the visit was LBP [27]. The physiotherapist-led model of care included: 1) initial assessment and risk assessment/stratification (using the Keele STarT Back tool [32]); 2) brief individualized intervention at first visit; 3) health services navigation; and 4) providing additional physiotherapy care for patients with an unmet need for treatment (i.e., if a patient was appropriate for physiotherapy care but did not have private insurance coverage) [27].

### Participants

#### Patients with LBP

Adults (18 years of age and over) with LBP who participated in the physiotherapist-led primary care model for LBP were eligible to participate in this study.

#### Primary care team members

Primary care team members (e.g., family physicians, nurse practitioners, dietitians, pharmacists, social workers, occupational therapists, administrators, and medical secretaries) who were part of primary care teams that were randomized to receive the physiotherapist-led primary care model for back pain were eligible to participate in this study.

### Sampling and recruitment

#### Patients with LBP

We used a purposive sampling technique to recruit patient participants with LBP who participated in the physiotherapist-led primary care model for LBP [33]. We aimed to get representation from: both men and women; patients at each risk category within the Keele STarT Back tool; those with acute and chronic LBP; and those who received ongoing care from the physiotherapist and those who received only an initial assessment and recommendations.

#### Primary care team members

We used a purposive sampling technique to recruit primary care team members who were randomized to receive the physiotherapist-led primary care model for LBP [33]. We aimed to get representation across disciplines within primary care teams, including interprofessional healthcare providers and those in administrative roles (e.g., managers, medical secretaries).

### Data collection

#### Semi-structured interviews

The first author (KV) conducted telephone semi-structured interviews with patient participants using a semi-structured interview guide [34, 35]. See Table 1 for questions from the interview guide.

**Table 1:** Questions from the semi-structured interview guide with patient participants

1.What were your expectations when you went to see the physiotherapist?
2.Could you please describe your experience with the physiotherapist?
3.Did you feel like the physiotherapist involved you to an appropriate degree in the decision making about treatment? Why? How did this experience compare with your expectations?
4.What were the good things about your experience with the physiotherapist?
5.What were the bad things about your experience with the physiotherapist?
6.At the first visit, the physiotherapist completed an assessment with you. This would have involved asking you questions and performing some physical tests, like bending and twisting, to learn about your LBP. How did you feel about the assessment?
7.At the first visit, your physiotherapist provided some education and a few exercises. Were you able to use the education and exercises provided to manage your LBP? What did you use? What did you not use? Why? What did you think about the education and exercises overall?
8.For people without insurance coverage for physiotherapy, the physiotherapist provided ongoing management for LBP in your doctor’s office. Did you receive any ongoing care from the physiotherapist? What did you think about approach? What did you think about the care you received?
9.What are your views of having the physiotherapist available to you as the first person you would see for LBP?
10.How did you feel the physiotherapist worked with your family doctor?
11.From your perspective, are there any draw backs to having the physiotherapist within the team?
12.How did having the physiotherapist in your team influence your use of other health care providers or services?
13.Did having the physiotherapist in the team change the outcome of your LBP?

*LBP* Low back pain

#### Focus group discussions

A co-author (CG) conducted in-person focus groups with primary care team member participants using a focus group script [36]. See Table 2 for questions from our focus group script.

**Table 2:** Questions from the focus group script with primary care team member participants

1.How did the integration of the physiotherapist within the team influence patient care?
2.Did the assessment or management for LBP change?
3.In what ways was it valuable to have the physiotherapist integrated within your team?
4.How confident were you in the assessment and management provided by the physiotherapist?
5.How satisfied were you with the integration of the physiotherapist within the team?
6.Did having the physiotherapist integrated within the team change your own workload or clinical processes?
7.Did having the physiotherapist integrated change referrals made or health care resources utilized?
8.Were there any drawbacks to having the physiotherapist integrated within the team?
9.What were some things that helped or would help make integrating the physiotherapist within the team easier?
10.Do you think having the physiotherapist integrated within the team changed patient outcomes, and if so, how?

*LBP* Low back pain

Interviews and focus group discussions were audio-recorded, transcribed verbatim, and reviewed for accuracy by the interviewer or focus group facilitator. We drew upon concepts of information power to determine when to stop data collection [37].

### Data analysis

To provide structure to our analysis process, we analysed interview and focus group data using Braun and Clarke’s approach to thematic analysis [38]. This included: 1) familiarization with the data, 2) generating initial codes, 3) searching for themes, 4) reviewing the themes, 5) defining and naming the themes, and 6) producing a report [38]. Consistent with an interpretive description approach, we considered questions like “so what?” and “what does this mean clinically” throughout our thematic analysis process. To assist with data management, transcripts were coded and stored within MAXQDA (Version 11. Berlin: VERBI Software; 2014).

As the first step of analysis, the first author (KV) reviewed each interview and focus group discussion transcript to familiarize himself with the data. Interview transcripts were analyzed first by a sub-set of the research team (KV, CD, AW, and JM). The first interview was coded independently by this sub-set of the team. We then had an in-person meeting to reach consensus on a preliminary coding scheme. The next four transcripts were then coded independently by two members of the research team (KV and AW) who then met in-person to reach consensus on codes. At this point, both coders determined an appropriate level of consistency in their approach to analysis. As such, the remaining interview transcripts were coded independently by one of these two members of the research team (KV or AW). After we coded our interview data, another sub-set of the research team (KV, CG, and JM) coded the focus group discussion transcripts. All three focus group transcripts were independently coded by this sub-set of the research team. After the three focus group transcripts were coded, this sub-set of researchers met in-person to discuss codes and agree upon a finalized coding structure. Next, we combined coded data from our interviews and focus group discussions. Specifically, the coded data were integrated into one file to allow the research team to identify commonalities across the interviews and focus group discussions. KV searched the coded data from the interviews and focus group discussions for themes and sub-themes. Once KV had developed initial themes and sub-themes, we met multiple times with a sub-set of the research team (KV, CD, CG, AW, and JM) to review our preliminary findings, and engage in reflexive discussion. Informed by these discussions, KV continued to define and name the themes and sub-themes through a collaborative and iterative process. Finally, we created a report describing our themes and sub-themes along with representative quotations.

### Trustworthiness

We used multiple strategies to enhance trustworthiness, including an audit trail, engaging multiple researchers in the coding process, participating in multiple in-person meetings with a sub-set of the research team throughout data analysis to engage in reflexive dialogue and discussion, and using supporting quotations to illustrate our findings [39].

## Results

Eighteen patients who participated in the new physiotherapist-led primary care model for LBP took part in a semi-structured interview. Interviews were between 30 to 60 min in duration. Most patient participants were women (10/18), had a median age of 52 years (range: 27–77 years), and reported experiencing LBP for a median of five months (range 1–124 months). In addition, 20 primary care team members participated in one of three focus group discussions (n = 8 family physicians, n = 3 office managers; n = 3 medical secretaries; n = 2 nurse practitioners, n = 2 registered nurses, n = 1 executive director, and n = 1 occupational therapist). Focus groups ranged from 45 to 60 min in duration. See Table 3 for additional details on patient participant demographic characteristics.

**Table 3:** Patient participant demographic information (*n* = 18)

Characteristic	Description
Gender, n	
Man	8
Woman	10
Age – years, median (range)	52 (27–77)
Duration of LBP in months, median (range)	5 (1–124)
Charlson Comorbidity Index [53], median (range)	1 (0–5)
Employment status	
Employed	9
Not employed	9
Household income per year, n	
$0–19,999	2
$20,000–39,999	5
$40,000–59,999	4
$60,000–79,999	3
$80,000–99,999	2
$ > 100,000	2
Keele STarT Back tool risk category, n	
Low	6
Medium	11
High	1
Physiotherapy care pathway, n	
Initial assessment with physiotherapist in primary care team and no further follow-up	2
Initial assessment and follow-up with physiotherapist in primary care team	10
Initial assessment with physiotherapist in primary care team and referral to community-based physiotherapist	5
Initial assessment and follow up with physiotherapist in primary care team plus referral to community-based physiotherapist	1

*LBP* Low back pain

**Fig. 1:**
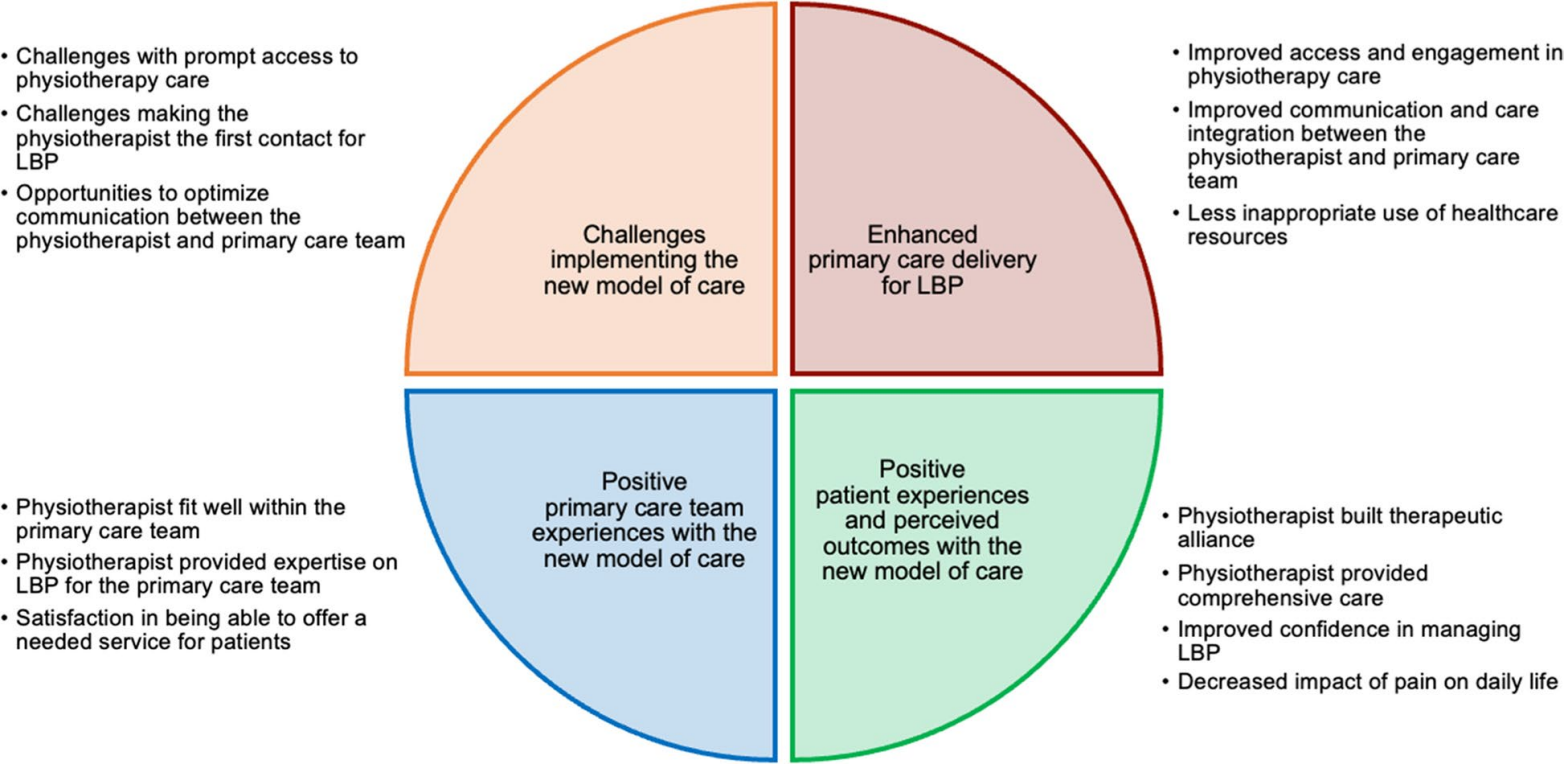
Themes/sub-themes related to perspectives towards the new physiotherapist-led primary care model for LBP

We constructed four themes and subsequent sub-themes relating to the new physiotherapist-led primary care model for LBP. See Fig. 1 for a visual representation of themes and sub-themes. For additional quotations, beyond those embedded within the text of this paper, see Supplemental File 1.

### Theme 1: Enhanced Primary Care Delivery for LBP

Participants reported that the new physiotherapist-led primary care model for LBP contributed to enhanced care delivery for LBP within the primary care setting.

#### Improved access and engagement in physiotherapy care

Participants described how the model of care contributed to improved access and engagement in physiotherapy care. Patient participants shared how they valued being able to access physiotherapy for their LBP with no out-of-pocket expense, as this helped to improve their ability to access physiotherapy care. Some participants described that they would not have been able to access this type of care if it was not available at their primary care site without personal cost. For example, one patient participant shared how finances are a barrier to accessing physiotherapy care that was no longer present with the new model of care:

*“[T]he majority of people don’t have enough money to pay for physiotherapy […] So having it at the office and having that for me is amazing.” INT-17*

Primary care team participants shared similar sentiments related to improved access to physiotherapy care for LBP. Participants described how many patients cannot access physiotherapy care due to limited private insurance coverage. They reported that this model of care helped to fill a gap by improving access for patients who would otherwise go without physiotherapy care:

*“I was happy about the increased access or that you could provide increased access to physio for patients that don’t have [insurance].” FG-1*

Participants also described how the model of care contributed to improved patient engagement and follow through in physiotherapy care for their LBP. Patient participants described how having the physiotherapy co-located within their primary care site was more convenient and a familiar space, which improved their likelihood and willingness to engage in physiotherapy care for their LBP. One participant shared that they may not have engaged in physiotherapy care if it was not co-located in their primary care site:

*“I may not have gone to a physiotherapist had I not had one in the doctors’ office.” INT-12*

Primary care team participants agreed that the model of care contributed to improved patient engagement in physiotherapy care for LBP for various reasons, such as easier booking, increased familiarity, and comfort accessing physiotherapy care within their primary care site. For example, participants described how many patients felt more comfortable accessing care within their office, which helped to increase patients’ likelihood of engaging in physiotherapy care for their LBP:

*“[…] having [the physiotherapist] within our office itself is a big bonus because um people feel a comfort here so and there’s more follow through [by patients].” – FG-2*

#### Improved communication and care integration between the physiotherapist and primary care team

Participants reported that the model of care contributed to improved communication and more integrated care between the physiotherapist and primary care team than would typically happen if the physiotherapist was not integrated directly within the primary care team. Patient participants described how this model of care allowed the physiotherapist and primary care team to easily share information and provide more integrated primary care for LBP:

*“[…] everybody’s on the same page […] having [a physiotherapist] that’s able to actually work with my doctor and work with me […] that for me personally was really really helpful.” INT-17*

Primary care team participants agreed that this model of care contributed to improved communication and more integrated care between the physiotherapist and primary care team when providing care for LBP. A shared electronic medical record was described by primary care team participants as a key facilitator to more integrated care between themselves and the physiotherapist. Primary care participants also spoke of the value of “hallway conversations” for informal sharing of information between the physiotherapist and primary care team, which was a benefit of this model of care. Participants described how improved communication allowed members of the primary care team to provide more integrated care with the physiotherapist for LBP. Many described how this model of care was superior to referring externally to a physiotherapist in the community, because there was improved communication, which allowed for the delivery of more consistent and integrated care for LBP:

*“[…] some [community] physiotherapists do send notes back to us [in primary care] but it doesn’t always feel like we’re part of the same team […] And then ah it gives me a much better idea of how to reinforce what [the physiotherapist] told them […] our role [in primary care] is to coordinate care within the health care system, well to some extent, for our patients, and that just makes it a lot easier for us to do if the person is actually part of our team.” FG-2*

#### Less inappropriate use of healthcare resources

Primary care team participants discussed how this model of care may reduce inappropriate use of healthcare resources for the management of LBP. For example, participants shared how having the physiotherapist integrated among the team to provide first contact care could contribute to less unnecessary diagnostic imaging for LBP, less unnecessary follow-up visits for LBP with primary care physicians, and decrease the number of inappropriate referrals for LBP (e.g., surgery). For instance, one participant shared how this model of care could decrease unnecessary diagnostic imaging:

*“Yea and requests for imaging I think will go down [with this model of care …] You know eventually a patient with low back pain would say – ok could you really need to do an MRI or something. And occasionally overtime, even when I don’t really think it’s necessary, it would end up happening just because of sort of wearing down over time.” FG-2*

Primary care team participants also shared how the model of care may contribute to fewer unnecessary follow-up visits with primary care physicians for LBP, as patient follow-up can happen with the physiotherapist:

*“[I]t was great because the physio just took over and did all the follow-ups [for low back pain]. So that was not somebody we had to put into the doctor’s schedules.” FG-3*

### Theme 2: Positive Patient Experiences and Perceived Outcomes with the New Model of Care

Participants described positive patient experiences and perceived outcomes with the new physiotherapist-led model of care for LBP.

#### Therapeutic alliance between the physiotherapist and patient

Patient participants described how a positive aspect of the model of care was that the physiotherapist built a strong therapeutic alliance. Participants described how the physiotherapist was genuine, present, respectful, and personable. Participants shared that the physiotherapist made them feel like a person (versus a patient or a number). One participant shared that the therapeutic alliance built with the physiotherapist allowed them to feel in charge of their care, while also supported:

*“That therapeutic alliance was there, right. So you kind of feel like you’re in charge but [the physiotherapist is] there to support you.” INT-2*

#### The physiotherapist provided comprehensive care

Patient participants agreed that a positive aspect of this model of care was that the physiotherapist provided comprehensive care. Participants described that the physiotherapist conducted a thorough assessment and spent the time to genuinely listen and hear their story. Participants reported that they valued that the physiotherapist conducted a thorough physical exam and was not rushed in their clinical visits. Participants also shared that the physiotherapist was thorough, providing tailored and whole person care that addressed specific day-to-day challenges with LBP. Participants described that they appreciated that the physiotherapist thoroughly described treatment plans and was open to answering questions. One participant emphasized that the time the physiotherapist spent with them allowed for an adequate assessment:

*“[The physiotherapist] took [their] time explaining things and did more tests to um you know to try to fully diagnose what was going on and um I thought that was [...] encouraging.” INT-3*

#### Improved confidence in managing LBP

Patient participants reported that a positive aspect of the model of care was that the physiotherapist helped to improve their confidence in managing LBP. Participants reported that the physiotherapist was reassuring, gave them hope, involved them in their care, and provided them with ownership over their health by providing them with active strategies to management their LBP, such as structured exercise. In combination, participants described how this helped to improve their confidence in managing their LBP:

*“I think it made me feel more confident because I could do something myself about [my low back pain].” INT-7*

#### Decreased impact of pain on daily life

Participants reported that a positive outcome of the model of care was that it helped to decrease the impact of pain on daily life. Participants described decreased pain intensity, improved sleep as a result of less pain, increased physical function, improved abilities at work, and improved overall enjoyment and quality of life. For example, one participant emphasized that their overall quality of life had improved and that their physical abilities had improved as a result of engaging in the model of care:

*“My overall quality of life for sure [has improved] because I could really only walk for about 10 or 15 minutes before I had to stop and kind of stretch out my back or do something. And now I can go, ah I walk for almost an hour, and ah you know it’s just starting to bother me then. So it’s made a huge difference for me.” INT-4*

### Theme 3: Positive Primary Care Team Experiences with the New Model of Care

Primary care team participants described positive experiences with the new physiotherapist-led model of care for LBP.

#### Physiotherapist fit well within the primary care team

Primary care team participants described how a positive aspect of the model of care was that the physiotherapist fit well within the primary care team. Participants emphasised the importance of the physiotherapist having a positive working relationship with other healthcare providers given the collaborative nature of the team-based primary care setting. For example, participants reported that they enjoyed working collaboratively with the physiotherapist, and that the physiotherapist was approachable, personable, and “fit” within the team:

*“I think [the physiotherapist] was quite personable [… and] seem[ed] to fit.” FG-2*

Ultimately, participants reported how the physiotherapist fit well and effectively integrated themselves amongst the existing primary care team:

*“Um but I found [the physiotherapist] to be ah quite personable. [They] seemed to integrate well with the team.” FG-3*

#### Physiotherapist provided expertise on LBP for the primary care team

Primary care team participants shared that a positive aspect of the model of care was that the physiotherapist provided expertise on LBP for the primary care team. Participants described how they would ask the physiotherapist clinical questions and benefited from seeking feedback on patient cases. Participants also shared how they were able to learn from the physiotherapist as a peer. One participant shared how the physiotherapist provided the primary care team with expertise on LBP, which complemented their skill set as a primary care provider:

*“It’s always nice to have a second set of eyes, somebody who’s specifically working within this component, ah where I can I do my assessment and the generalist stuff [as a family physician] and [the physiotherapist is] the specialist in this area.” FG-1*

Participants also reported how the physiotherapist was able to leverage their expertise on LBP to provide peer-to-peer education to other members of the primary care team:

*“Yea just [the physiotherapist’s] clinical expertise and peer education from my perspective was great.” FG-3*

#### Satisfaction in being able to offer a needed service for patients

Primary care team participants reported satisfaction in being able to offer a needed service (e.g., physiotherapy care) for patients within their primary care team. Participants described how it felt good having a physiotherapist integrated within the primary care team as it helped to create a “medical home” for their patients:

*“I like the idea of the medical home and this [model of care] obviously enriches the medical home for our patients.” FG-3*

### Theme 4: Challenges Implementing the New Model of Care

Participants described challenges when implementing the new physiotherapist-led primary care model for LBP.

#### Challenges with prompt access to physiotherapy care

Some participants described challenges with prompt access to see the physiotherapist. For example, one patient participant described how they had a challenge scheduling an appointment time to see the physiotherapist due to limited options:

*“There was a very short window of opportunity to see the physiotherapist ah because [they were] so busy. Ah so there was only a couple of times during his week where [the physiotherapist] could see me. And it just so happened that they fell on the days that I do have my children […] unfortunately, because of the scheduling it made it difficult.” INT-2*

Some primary care team participants also described challenges with prompt access for patients to see the physiotherapist. One participant described how the wait to see the physiotherapist at their site was longer than the wait to see a family physician. This participant linked this to the fact that the physiotherapist was providing care at multiple primary care sites as part of the pilot study:

*“I think the only draw-back that I can think of is that we did not have [the physiotherapist here] 5 days a week because unfortunately, you know, we’re sharing [their] time with other clinics. And the times that we were allotted don’t always work for the patients.” FG-1*

#### Challenges making the physiotherapist the first contact for LBP

Primary care team participants described some challenges with making the physiotherapist the first contact for LBP as part of the model of care. Challenges related to booking patients directly in to see the physiotherapist. For example, one primary care team participant described that when patients called to make an appointment, they did not always mention LBP as the primary reason for their visit to the receptionist:

*“I think it was a little difficult um like booking wise […] I don’t think patients just called and said they were coming in for low back pain. So um most of our referrals I think were from the physician and not directly from the front staff.” FG-2*

Despite this, this primary care team participants hypothesized that if the model of care was implemented for a longer time period, and patients knew it was an option for them to book directly in with the physiotherapist, these challenges may subside:

*“But if the physiotherapist was incorporated within the team [over the long term] and patients knew about it […] I think [this model of care] would work well.” FG-2*

Although there were some challenges making the physiotherapist the first point of contact, most patient participants agreed that seeing a physiotherapist as the first point of contact for LBP was an appropriate care pathway:

*“I don’t think that people should be seeing the general practitioner for ongoing problems with low back pain or any other kind of pain. I feel very strongly that that should be handled first and directly by someone [like a physiotherapist] that specializes in that issue.” INT-17*

#### Opportunities to optimize communication between the physiotherapist and primary care team

Some participants described opportunities to optimize communication between the physiotherapist and broader primary care team members. Some primary care team participants described that communication would be improved between themselves and the physiotherapist if there was dedicated time for collaboration on patient care (e.g., patient rounds) and if the physiotherapist was on-site more (e.g., during lunch hour) to promote more informal conversation and sharing of information. Furthermore, one primary care team participant described how the physiotherapist’s documentation could be more seamlessly integrated within the electronic medical record at their primary care site:

*“I would like the [physiotherapist’s documentation] to be typed in the chart versus a scanned document that’s not searchable.” FG-1*

## Discussion

From the perspective of patients and primary care teams, a new physiotherapist-led primary care model for LBP contributed to enhanced primary care delivery for LBP, positive patient experiences and perceived outcomes, and positive primary care team experiences. We also identified specific challenges experienced when implementing the new service delivery model, including issues related to timely access, difficulties making the physiotherapist the first contact for LBP, and opportunities to optimize communication between the physiotherapist and broader primary care team. These findings complement existing qualitative research on perspectives towards [24, 25] and proposed models [26] of first-contact physiotherapy primary care by providing in-depth perspectives of patient and primary care teams based on their experiences with a new physiotherapist-led primary care model for LBP in a region of Ontario, Canada. To our knowledge, this is the first study to specifically explore patient and primary care team perspectives towards a physiotherapist-led primary care model for LBP in the Canadian context. In this model of care, a physiotherapist was available as the first point of contact who could provide additional care for patients with an unmet need for treatment.

Participants described that the new physiotherapist-led primary care model contributed to enhanced primary care delivery for LBP, including improved communication and more integrated care between the physiotherapist and other members of the primary care team. This aligns with previous research that has indicated that co-location of interprofessional healthcare providers and family physicians in primary care can improve collaboration and care integration [40]. Importantly, this finding aligns with calls to action for more integrated care for LBP [9] and suggests that within clinical practice, there is value in having a physiotherapist integrated directly within the primary care team to provide clinical care. We also found that participants described that this new service delivery model may contribute to less inappropriate use of healthcare resources, such as unnecessary diagnostic imaging for LBP. These are interesting findings, given that unnecessary diagnostic imaging for LBP is costly [41] and can negatively impact patient beliefs about LBP [42]. This finding suggests that physiotherapist-led primary care models may contribute to more guideline adherent primary care management of LBP, given that practice guidelines recommended that diagnostic imaging be used judiciously for patients with LBP [43, 44].

From the perspective of patients and primary care team members, the new service delivery model contributed to positive patient experiences and perceived outcomes. For example, participants described that the physiotherapist spent the time to build therapeutic alliance by listening and supporting self-management for people with LBP. This is a compelling finding given that many patients with pain experience dismissal and judgement by healthcare providers in primary care [45]. This suggests that all healthcare providers within primary care settings should place specific emphasis on building therapeutic alliance to improve patient experiences when seeking care for LBP. Furthermore, patient participants described how they had confidence managing their LBP as a result of receiving support to engage in physiotherapy care, such as physical activity and therapeutic exercise. Given the barriers that people with pain can face when participating in physical activity and exercise [46], and a desire for healthcare providers to provide individualized support when engaging in physical activity and exercise [47], this model of care may be a viable approach to promote guideline adherent care (e.g., promoting engagement in physical activity and exercise) [43, 48]. Recent evidence suggests that usual care for LBP within primary care does not align well with clinical practice guidelines, with only 20% of patients with LBP receiving evidence-based information from their primary care physician [49]. Therefore, these findings further support the notion that physiotherapist-led primary care models for LBP may provide a viable option to improve the implementation of guideline-adherent management of LBP within the primary care setting.

Primary care team participants described positive experiences with the new physiotherapist-led primary care model for LBP. The fact that primary care teams expressed positive experiences with this new service delivery model is an encouraging finding given an increasing emphasis on the importance of healthcare provider experiences, as described within the Quadruple Aim framework in Canada [50]. In particular, participants described how the physiotherapist provided expertise on LBP for the primary care team, and that they valued being able to learn peer-to-peer from physiotherapist. Given the fact that family physicians receive limited training on musculoskeletal disorders [51], this finding suggests that this model of care may help to complement primary care physician skillsets, and ultimately, lead to more comprehensive primary care services for LBP. Furthermore, this finding emphasizes that physiotherapists within primary care should embrace opportunities to support and assist their interprofessional colleagues in the management of musculoskeletal conditions, such as LBP.

Participants also highlighted challenges implementing the new model of care. Challenges with timely access, booking patients in to the see the physiotherapist as the first point of contact, and opportunities to optimize communication between the physiotherapist and the primary care team (e.g., importance of seamless integration and use of a shared electronic medical record) were described. These are relevant findings for future research related to this new service delivery model. For example, it may be important to consider opportunities for improved communication between the physiotherapist and the primary care team as collaborative and responsive communication between healthcare providers is an important component of interprofessional collaboration [52]. In future research related to this model of care, it may be valuable to integrate processes for regular communication between the physiotherapist and the primary care team as a core element of the model of care. Additionally, these findings suggest that within day-to-day clinical practice, specific efforts should be made to support optimal communication between physiotherapists and other members of the primary care team.

## Limitations

This study was nested within a pilot cluster randomized controlled trial [27] where significant resources ensured the fidelity of the physiotherapist-led primary care model for LBP. As such, despite being a pragmatic trial design, it is possible that the intervention was more standardized than would happen in typical clinical practice. This may have positively impacted what patient and primary care team participants reported, which may impact the transferability of these findings into the ‘real world’ clinical environment. Furthermore, the physiotherapist-led primary care model for LBP was implemented within team-based models of primary care (i.e., Family Health Teams and Community Health Centres) in a region of Ontario, Canada. Therefore, the transferability of our findings to other models of primary care (e.g., solo family physician practices) and geographic contexts is not clear.

## Conclusion

We identified four themes and subsequent sub-themes related to patient and primary care team perspectives towards their experiences with a new physiotherapist-led primary care model for LBP, including: enhanced primary care delivery for LBP, positive patient experiences and perceived outcomes with the new model of care, positive primary care team experiences with the new model of care, and challenges implementing the new model of care. Participants described that the model of care contributed to positive experiences for patients, primary care teams, and potential impacts for the healthcare system. Challenges to be addressed when scaling up the new model of care include: timely access to physiotherapy care, difficulties making the physiotherapist the first contact for LBP, and opportunities to optimize communication between the physiotherapist and other members of the primary care team. Our results demonstrate that a new physiotherapist-led primary care model for LBP may be a viable approach to support more integrated and guideline adherent management of LBP in primary care.

## Supplementary Information

**Additional file 1.** Additional supporting quotations

## Data Availability

The datasets generated and/or analyzed during the current study are not publicly available due to limitations of ethical approval involving the patient data and anonymity but are available from the corresponding author on reasonable request. **Ethical approval and consent to participate.** Ethics approval was obtained from the Queen’s University Health Sciences and Affiliated Teaching Hospitals Research Ethics Board in Kingston, Ontario, Canada (Approval Number: 6024354). All methods were performed in accordance with relevant guidelines and regulations. All participants provided written informed consent prior to participating in this research.

## Abbreviations

LBP: Low back pain
