# Supplementary material for: Implementing a new physiotherapist-led primary care model for low back pain: a qualitative study of patient and primary care team perspectives
Source: BMC Prim Care. 2022 Aug 11;23:201. doi: 10.1186/s12875-022-01817-5 (PMC9367061; doi:10.1186/s12875-022-01817-5)
Supplement: Supplementary file 1 — Additional file 1. Additional supporting quotations [file 12875_2022_1817_MOESM1_ESM.docx]

**Additional Supporting Quotations**

| **Theme/sub-themes** | **Supporting quotations** |
| --- | --- |
| *Theme 1: Enhanced primary care delivery for LBP* | |
| Improved access and engagement in physiotherapy care | “I asked my doctor and she’s the one who referred me to the [physiotherapist] because it was upstairs […] So I thought that was really convenient […] I’m familiar with the building and I thought that was really easier for me than to go all the way downtown.” INT-1  “Ah we don’t have [private] coverage so like I said ah I wouldn’t have sought [physiotherapy] out unless it was, you know dire. Um so it was very very nice and convenient to have access to that right away and so yea I couldn’t have paid for [physiotherapy] out of pocket otherwise.” INT-10  “[S]o the nice thing was, you’ve seen the doctor, you get booked – and so people did it. And that that compliance part, that you know, following the plan I think it really made a huge difference [that the physiotherapist was] inside the team.” FG-1 |
| Improved communication and care integration between the physiotherapist and primary care team | “I think one of the problems in in any medical field is the time gap between the generalist, the specialist, call it whatever you want. There’s always something that’s lost in the translation. So I think the fact of having [a physiotherapist] in the office is just a real positive.” INT-9  “I also like the um documenting and how [the physiotherapist] actually um kind of reviewing [their] findings. And when a patient came back after seeing [the physiotherapist], it was really nice to pull that up [in the electronic medical record] and kind of see, ok, this is consistent with what [the physiotherapist] saw.” FG-1 |
| Less inappropriate use of healthcare resources | “I think it’s a more targeted appropriate use of resources and awareness of some resources that I wasn’t aware of.” FG-2  “I think I had probably two cases where I was considering doing imaging and then [the physiotherapist] assessed and agreed with them, so in some cases it was also more, maybe you’re not saving resources but it’s more, like rationale for doing the test because now you have two people saying that this test is warranted in this patient. So yes I think you do avoid some, but in the cases where it’s needed I think you have more support into why you’re ordering it.” FG-2  “And so having [the physiotherapists] complete assessment, [their] level of concern about it, um means that I’m much more likely to – like this person might need surgery kind of. I’m much more likely to appropriately refer this person for surgery if X, Y, Z happens.” FG-2 |
| *Theme 2: Positive patient experiences and perceived outcomes with the new model of care* | |
| Physiotherapist built therapeutic alliance | [The physiotherapist] made me feel like I was important as a person not a body with a problem.” INT-5  “[The physiotherapist] treated me like a person not a patient I think and that’s a part of the problem when it comes to health care.  Too much treating people like patients and not really giving enough control for the person who’s in pain.” INT-6  “Yeah but at the same time [the physiotherapist was] just a real genuine positive person that doesn’t go and treat their patient like a patient.” INT-17 |
| Physiotherapist provided comprehensive care | “I think [this model of care was] a little bit more in-depth because I had more time with [the physiotherapist] than I than I probably generally would be with a another physiotherapist because the physiotherapists that I was seeing, you know, you go in there, you hop on a table, they put pins in you, then they leave the room. Whereas this physiotherapist was more, you know, discussing the challenges of your life and listening to you and having a better understanding of of the restrictions that you have in your life.” INT-2  “It wasn’t just a sheet of paper with some exercises. [The physiotherapist] actually went through them with me, so that was really helpful I think.” INT-4  “[The physiotherapist] was very thorough.” INT-12 |
| Improved confidence in managing LBP | “I felt confident that I could help myself.” INT-1  “Oh I think I had a lot more confidence in um managing the back pain because of being, you know, what I’d learned that day […] And it it gave me more reassurance that I was going to be able to get through the next few months.” INT-10  “Well just now having the knowledge of knowing what to do certainly promotes confidence.” INT-14 |
| Decreased impact of pain on daily life | “Well it has helped. I have had less pain and I think I’m improving.” INT-1  “It brought it it brought me back some of my functionality you know. And it brought me back some you know some of the positive you know the positive aspects of your life, you you being able to enjoy you know things.” INT-2  “I don’t have back pain through the night. I’m not as restless as I used to be. And at my workplace, standing for long periods of time, in one place or another sort of thing, it’s not there. [The pain is] almost gone really.” INT-15  “That’s been one of the biggest things because I do a construction job, I can feel myself getting stronger and especially in my back. Um because like before that, stuff I wasn’t able to lift and work with very easily. Now I can almost pick up with one arm, you know, like I can feel the strength in me and the muscles that I haven’t been using um starting to come into play.” INT-16 |
| *Theme 3: Positive primary care team experiences with the new model of care* | |
| Physiotherapist fit well within the primary care team | “[The physiotherapist] just kind of seamlessly fit in.” FG-1  “I think [the physiotherapist] enjoyed being here as much as we enjoyed having [them] here. And um we had, you know, fun moments um where you know a joke and a laugh and then we’d all move on and work. And so it was nice. [They] felt like someone that we’d [hire], and we’re very particular about who we hire for our office. And [they] felt like someone that we would intentionally pick. There was no awkwardness or any uncomfortable moments.” FG-1 |
| Physiotherapist provided expertise on LBP for the primary care team | “I would actually go to [the physiotherapist] and ask [them] advice. And I would send [them] copies of like MRIs and say – what do you think.” FG-1  “I thought it was a great opportunity to have the physiotherapist on site and ah and have [their] feedback on the cases. I thought it was great.” FG-2 |
| Satisfaction in being able to offer a needed service for patients | “So I think we were getting better care of patients by having that service in-house.” FG-1  “[…] it was exciting to be able to offer something to people [with LBP].” FG-1  “[…] it meant I had another, something to offer people who I thought needed physiotherapy but couldn’t afford it or had no coverage for it.” FG-2 |
| *Theme 4: Challenges implementing the new model of care* | |
| Challenges with prompt access to physiotherapy care | “[T]he first appointment that I had from [the physiotherapist] was 10 days from my original ah issue. And in that 10 days, by the time [the physiotherapist] saw me I was about 85-90% back to normal. Ah had I, with his information earlier, I could have been at that days earlier.” INT-6  “I just found because of the time frame for booking [with the physiotherapist] got, like at some point we were booking like a month out, right. So I think that that part didn’t meet my expectations. Just the availability.” FG-1  “I will also say, because of my time when I did have someone that was LBP in my schedule and the physiotherapist was here, I’d often did go to the secretaries and just be like – did we ask about the study or, because I have short visits and it’s quick quick to do and I knew that they’d get a thorough assessment. And I think a couple of times they were in my schedule, although [the physiotherapist] was here, because [the physiotherapist] was already full and people don’t want to wait.” FG-3 |
| Challenges making the physiotherapist the first point of contact for LBP | “I fully expected that patients would be diverted to physio rather than to us, which actually didn’t happen.” FG-1  “Um I know most of our referrals [to the physiotherapist] were direct referrals from the physician rather than when patients called in to ah and said they had back pain and we could just book them in.” FG-2  “[I]f people are used to for many many years that when they get acute pain they go see their doctors first, I think that would be a hard switch for mentality to bypass the doctor. Like if this is how they’ve been doing it for the last 30 years that they’ve been with their doctor. It’s a change in process that will take time and more time to make a difference.” FG-3 |
| Opportunities to optimize communication between the physiotherapist and primary care team | “Um I think what would have been ever more beneficial would have been to have had [the physiotherapist] here over lunch one day a week, or whatever, because we gain so much when from other team members. You know those informal discussions that happen I think is just as valuable as the formal stuff that happens, and we learn from that.” FG-1  “So if you’re not aware that a patient has had an encounter you don’t know that, like we don’t read other people’s notes unless they’re flagged to us [within the primary care team]. So I did have a couple of people that were seeing [the physiotherapist] without my knowledge and then [the physiotherapist] asked me about them, you know, a couple of visits later and I didn’t know.” FG-2  “So the direct feedback I thought was really useful, like having [the physiotherapist] come down. But sometimes it’s hard in the middle of a clinic to be able to be receptive to that. Like, if you’re running late and then and trying to discuss. So maybe having more time to actually, like have like a debrief like rather than between.” FG-2 |

LBP=low back pain.
